# Hepatitis C virus infection inhibits a Src-kinase regulatory phosphatase and reduces T cell activation *in vivo*

**DOI:** 10.1371/journal.ppat.1006232

**Published:** 2017-02-24

**Authors:** Nirjal Bhattarai, James H. McLinden, Jinhua Xiang, M. Meleah Mathahs, Warren N. Schmidt, Thomas M. Kaufman, Jack T. Stapleton

**Affiliations:** 1 Research and Medical Services, Iowa City Veterans Affairs Medical Center, Iowa City, Iowa, United States of America; 2 The Department of Internal Medicine, University of Iowa, Iowa City, Iowa, United States of America; 3 The Department of Microbiology, University of Iowa, Iowa City, Iowa, United States of America; The Scripps Research Institute, UNITED STATES

## Abstract

Among human RNA viruses, hepatitis C virus (HCV) is unusual in that it causes persistent infection in the majority of infected people. To establish persistence, HCV evades host innate and adaptive immune responses by multiple mechanisms. Recent studies identified virus genome-derived small RNAs (vsRNAs) in HCV-infected cells; however, their biological significance during human HCV infection is unknown. One such vsRNA arising from the hepatitis C virus (HCV) E2 coding region impairs T cell receptor (TCR) signaling by reducing expression of a Src-kinase regulatory phosphatase (PTPRE) *in vitro*. Since TCR signaling is a critical first step in T cell activation, differentiation, and effector function, its inhibition may contribute towards HCV persistence *in vivo*. The effect of HCV infection on PTPRE expression *in vivo* has not been examined. Here, we found that PTPRE levels were significantly reduced in liver tissue and peripheral blood mononuclear cells (PBMCs) obtained from HCV-infected humans compared to uninfected controls. Loss of PTPRE expression impaired antigen-specific TCR signaling, and curative HCV therapy restored PTPRE expression in PBMCs; restoring antigen-specific TCR signaling defects. The extent of PTPRE expression correlated with the amount of sequence complementarity between the HCV E2 vsRNA and the PTPRE 3’ UTR target sites. Transfection of a hepatocyte cell line with full-length HCV RNA or with synthetic HCV vsRNA duplexes inhibited PTPRE expression, recapitulating the *in vivo* observation. Together, these data demonstrate that HCV infection reduces PTPRE expression in the liver and PBMCs of infected humans, and suggest that the HCV E2 vsRNA is a novel viral factor that may contribute towards viral persistence.

## Author summary

The mechanism by which hepatitis C virus (HCV) establishes persistent human infection is complex and incompletely understood. Recent studies identified virus-derived small RNAs (vsRNAs) in HCV-infected cells; however, their biological significance is unclear. One HCV vsRNA arising from the E2 coding region reduces expression of a Src-kinase regulatory phosphtase (PTPRE) both in hepatocytes and lymphocytes *in vitro*, and leads to impaired T cell function. Here, we show that PTPRE expression is reduced in liver tissues and peripheral blood mononuclear cells (PBMCs) obtained from HCV-infected humans. Furthermore, serum from HCV infected individuals reduced antigen-specific TCR signaling, and curative anti-HCV therapy restored PTPRE expression in HCV-infected humans coincident to rescuing antigen-specific TCR-signaling defects. Transfection of a hepatocyte cell line with HCV genomic RNA or synthetic vsRNA duplexes inhibited PTPRE expression, recapitulating the *in vivo* observations. Together, these data suggest that HCV genomic RNA is processed into short, regulatory HCV RNA sequences that regulate PTPRE levels in HCV-infected humans, contributing to HCV immune evasion *in vivo*.

## Introduction

Hepatitis C virus (HCV) persistently infects more than 120 million people globally, and chronic viremia frequently leads to cirrhosis and hepatocellular carcinoma [1–8]. Although numerous factors appear to contribute to viral persistence, the mechanisms by which HCV evades immune responses are incompletely understood. Prior studies found that HCV-infection is associated with reduced T cell function *in vitro*, impaired HCV-specific intrahepatic and peripheral T cell response *ex vivo*, delayed onset of HCV-specific humoral and cellular immunity *in vivo*, and impaired immune responses to HBV and adenoviral vaccination [2, 8–20].

We recently reported that incubation of peripheral blood mononuclear cells (PBMCs) with plasma derived HCV, infectious cell culture derived HCV, and serum exosomes containing HCV RNA reduced IL-2 release and CD69 upregulation by T lymphocytes following activation through the T cell receptor (TCR) [11]. Expression of the HCV envelope (E2) coding RNA in Jurkat cells was sufficient to reduce TCR signaling, and to reduce phosphorylation of the lymphocyte-specific, protein tyrosine Src kinase (Lck). Deletion mutagenesis of HCV E2 RNA reducing Lck activation demonstrates that a short RNA region is sufficient to reduce TCR signaling [11]. This E2 RNA sequence contains a conserved 8 base region complementary to two sites in the 3’UTR of the Src regulatory phosphatase PTPRE (protein tyrosine phosphatase receptor epsilon) [11].

PTPRE activates signaling by Src family tyrosine kinases [21–23], and previous studies demonstrate that inhibition of Src-kinase signaling promotes HCV replication [24, 25]. Expression of HCV vsRNA is sufficient to reduce PTPRE protein levels in Jurkat cells, and mutation of conserved residues in the HCV E2 short RNA region restore PTPRE levels and TCR-mediated Lck activation [11]. PTPRE specificity was confirmed by placing the PTPRE 3’UTR sequences after GFP, and showing that HCV E2 expression regulated GFP expression in this system. Furthermore, replacement of the PTPRE targeting sequence in HCV E2 with a sequence targeting CXCR4 restored PTPRE levels and reduced CXCR4 expression. Thus, a virus (HCV E2) RNA-derived, short RNA (vsRNA) regulates PTPRE and reduces TCR signaling *in vitro* [11].

Although DNA viruses and retroviruses generate functional vsRNAs [26, 27], the ability of strictly cytoplasmic RNA viral genomes to be processed into functional vsRNAs is controversial [28–30]. Short RNA species are found in HCV and other cytoplasmic RNA virus infected cells [11, 31–35]; however, there are no data demonstrating that these vsRNAs are functional during human infection. Here, we expand the previous *in vitro* characterization of the HCV vsRNA effect on TCR signaling by showing that synthetic HCV genomic and vsRNA regulate PTPRE via one of the two potential target sites with complementarity within the PTPRE 3’UTR, and that HCV regulates TCR and PTPRE expression in human liver tissue and PBMCs during HCV infection. Importantly, curative HCV therapy restored both PTPRE levels and T cell activation following TCR stimulation. The data provide the first *in vivo* evidence of a functional vsRNA generated from the HCV genome, and identify PTPRE as a novel cellular factor regulating T cell activation.

## Results

### HCV RNA-containing serum inhibits antigen-specific TCR signaling

**Fig 1:**
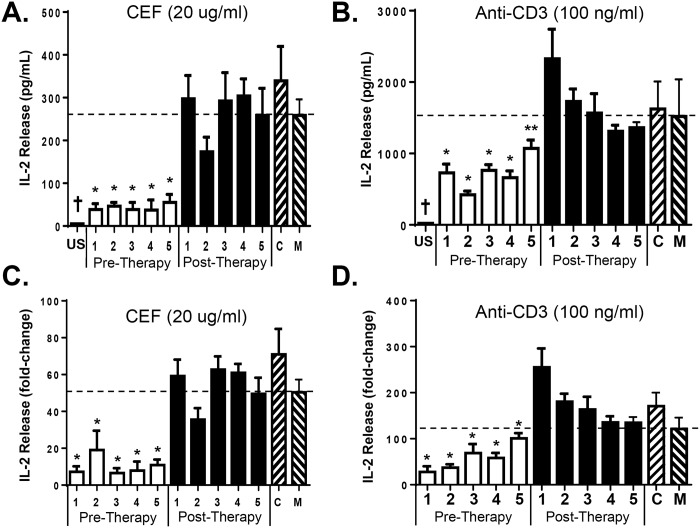
HCV RNA-containing sera inhibit antigen-specific and anti-CD3-mediated T cell receptor signaling. Healthy donor PBMCs were incubated in serum obtained before or following curative HCV therapy for 24 hrs, stimulated with a pool of antigenic peptides from CMV, EBV, and influenza virus (CEF; A) or anti-CD3 (B), and IL-2 measured 16 hrs later. US = unstimulated. C = control, these cells were incubated in serum pooled from five HCV-uninfected individuals. M = no serum control. * p<0.01, ** p< 0.05 compared to post-treatment samples and controls, † US vs. all other samples. Data in panels A and B represent results from three replicates in a single donor. Experiments were repeated in two additional healthy donor PBMCs and the combined fold-change in IL-2 release for all three donors following CEF (C) or anti-CD3 (D) for the same sera samples is shown.

To determine if HCV RNA-containing sera inhibits antigen-specific TCR signaling, PBMCs from three healthy blood donors were incubated in sera obtained from 5 HCV infected donors before and following curative HCV therapy. Sera pooled from 5 HCV uninfected individuals served as the negative control. Following overnight incubation, cells were stimulated with either viral T-cell antigenic peptides from CMV, EBV, and influenza (CEF peptides; Anaspec) or anti-CD3 antibody. Incubation of PBMCs in HCV infected patient serum reduced, but did not abolish IL-2 release following antigen-specific T cell receptor stimulation (representative donor PBMCs in Fig 1A). Following curative HCV treatment, IL-2 release by cells incubated in the five treated HCV patients was not different than IL-2 released by cells incubated in pooled sera from five HCV-negative subjects or in cells that were not incubated in human serum (Fig 1A). HCV RNA positive serum also reduced TCR signaling induced by anti-CD3 stimulation, and as expected anti-CD3 was more potent in inducing IL-2 than the antigen-specific stimulation (Fig 1B). Although markedly different concentrations of IL-2 were released by PBMCs obtained from different blood donors following TCR stimulation, the fold-change in IL-2 following TCR stimulation followed the same pattern of inhibition by HCV RNA-containing sera. Following curative therapy, the same patient’s sera did not inhibit IL-2 release (Fig 1C and 1D).

**Fig 2:**
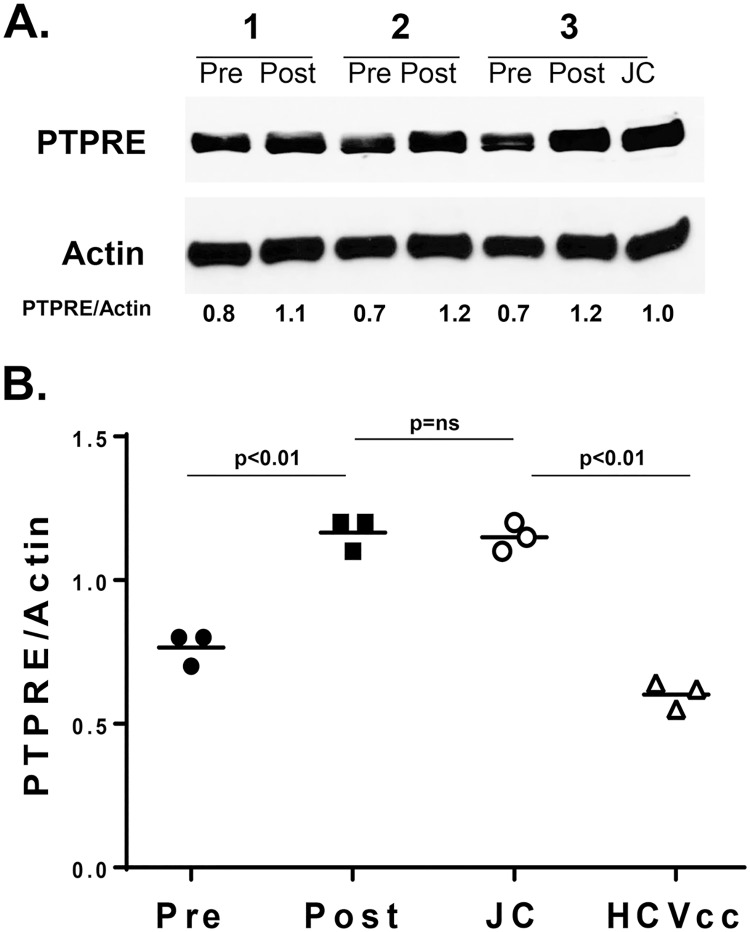
Serum and HCVccs regulate PTPRE in a human T cell line. Jurkat cells (2 x 10^6^) were incubated with serum obtained from three HCV-infected patients obtained prior to (Pre) or following (Post) curative HCV treatment for 24 hours. Total cellular PTPRE and actin expression was measured by immune blot (A). Alternatively, Jurkat cells were incubated in a preparation of Huh7.5 cell culture generated, infectious HCV particles (HCVccs), or in media (JC), and relative expression compared to actin determined. Data represent results from three independent experiments.

Previous studies found that serum from HCV-infected individuals also regulates TCR-mediated IL-2 release in a CD4+ T cell line (Jurkat cells) [11]. Jurkat cells were incubated in HCV RNA-positive sera before or following direct anti-HCV therapy, and PTPRE expression was measured by immune blot (Fig 2A). PTPRE was reduced in Jurkat cells incubated in serum from HCV infected people prior to treatment, but this reduction was lost following treatment (Fig 2B). Furthermore, Jurkat cells incubated in Huh7.5 cell culture-derived infectious HCV particles (HCVccs) also reduced PTPRE expression relative to that expressed in Jurkat cells incubated in post-treatment serum, or in control Jurkat cells incubated in a pool of HCV negative donors (Fig 2B). Together, these data show that serum from HCV-infected individuals reduces both TCR-signaling as measured by IL-2 release and PTPRE expression. Further, HCVcc particles lacking other serum factors similarly reduce PTPRE expression, and as previously shown, reduces TCR signaling [11].

### Synthetic HCV RNA is sufficient to regulate PTPRE expression

Stable expression of a short region of HCV E2 RNA in Jurkat cells negatively regulates TCR signaling, PTPRE expression, and Lck phosphorylation following TCR activation [11]. Here, we examined transfection of *in vitro* transcribed, full-length, infectious HCV RNA or a synthetic RNA duplex comprised of the HCV vsRNA sequence to determine if transfection of HCV RNA was sufficient to regulate PTPRE expression.

Bioinformatic analyses identified several genes predicted to be targeted by the HCV vsRNA based on the putative seed sequence, including PTPRE, Vesicle-associated membrane protein-A (VAPA), and growth factor receptor-bound protein 2 (Grb2) [36–38]. Like PTPRE, the VAPA 3’UTR contains two sequences with at least 7 bases complementary to a conserved 8 nt HCV RNA sequence within the HCV vsRNA, while Grb2 contains one such target site. VAPA is a proviral factor required for HCV replication, thus reducing its expression would be deleterious for HCV, and Grb2 is a positive regulator of Src kinase signaling, thus inhibition could contribute to impaired TCR signaling [39–42].

**Fig 3:**
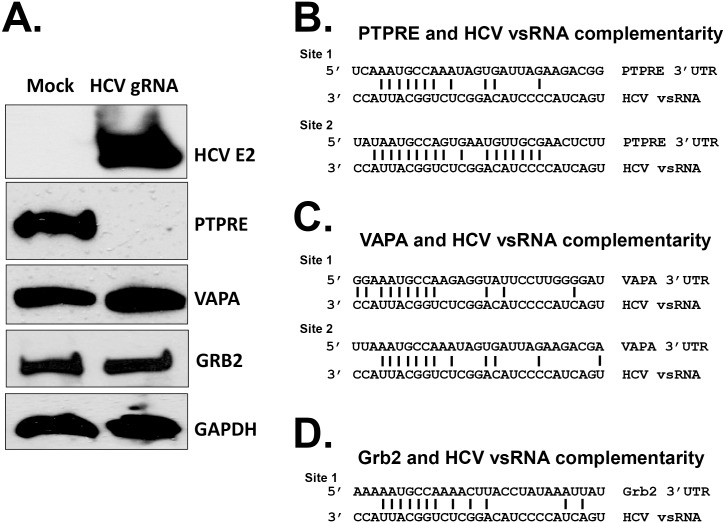
Hepatitis C virus genomic RNA selectively inhibits PTPRE expression. Huh 7.5 cells were mock transfected or transfected with full-length, *in vitro* transcribed HCV genomic RNA (gRNA) (A). HCV E2, PTPRE, VAPA and Grb2 protein levels were analyzed 96 hours later by immunoblot analysis (A). GAPDH served as the loading control. PTPRE (B), VAPA (C), and Grb2 (D) have 7 to 8 nt sequences complementary to the highly conserved HCV 8 nt sequence. There is 38% complementarity between the HCV RNA sequence examined and VAPA, Grb2, and Site 1 of PTPRE, and 56% complementarity with Site 2 of PTPRE. PTPRE, VAPA and GAPDH expression were measured 96 hours post transfection by immunoblot analysis. Experiments were performed three times with consistent results.

Because HCV is hepatotropic, and due to poor transfection efficiency of Jurkat cells, we transfected the HCV permissive hepatocyte cell line Huh 7.5 with full length HCV genomic RNA (HCVgRNA) transcribed from an infectious clone (kindly provided by Drs. Rice and Wakita) [43]. PTPRE levels were reduced in the HCVgRNA transfected cells compared to sham transfected cells. In contrast, VAPA and Grb2 expression levels were not altered (Fig 3A). Alignment of the 29 base HCV vsRNA sequence identified in HCV infected Huh7.5 cells by Andrew Fire’s laboratory [31], found 38% complementarity between the HCV vsRNA sequence and site 1 of the PTPRE 3’ UTR (Fig 3B), both sites of VAPA 3’UTR (Fig 3C), and the Grb2 3’ UTR sequence (Fig 3D). However, there was 56% complementarity between the vsRNA sequence and site 2 on the PTPRE 3’ UTR (Fig 3B). Furthermore, all of the target sequences contained 7 bases complementary to the conserved 8 base HCV sequence except site 2 of PTPRE. PTPRE 3’UTR site 2 contained 8 bases complementary to the vsRNA sequence of all 627 isolates listed in the Los Alamos database (http://hcv.lanl.gov/content/sequence/HCV/ToolsOutline.html). In addition, PTPRE Site 2 was complementary to 9 HCV E2 conserved bases in the majority of these isolates (Fig 3). These results suggest that the number of bases within the target sequence complementary to the HCV sequence and the flanking HCV sequences may contribute to target gene specificity.

**Fig 4:**
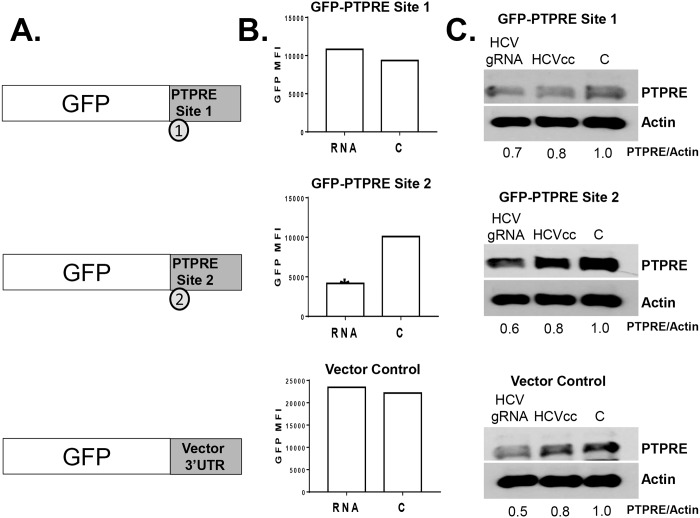
PTPRE 3’UTR site 2 required for HCV serum regulation of gene expression. HEK 293 cells stably expressing GFP containing PTPRE site 1 (A), site 2 (B), or neither (vector control; VC) after GFP were transfected with synthetic, genome-length HCV RNA (HCV gRNA), transfection reagent control (C), or incubated in cell culture infectious HCV particles (HCVccs). GFP expression was measured by flow cytometry (B), and PTPRE expression was measured in by immune blot analyses (C). * = p<0.05. Experiments were performed three times with consistent results.

To determine which of the two PTPRE 3’UTR target sites interacted with the HCV vsRNA, the PTPRE site 1 sequence and the PTPRE site 2 sequence were independently inserted into the 3’UTR region of GFP as illustrated (Fig 4A). These plasmids and the parent GFP expression plasmid were used to generate human embryonic kidney (HEK) 293 cell lines stably expressing GFP as previously described [11]. Each cell line was transfected with a synthetic, genome-length, HCV RNA transcript or the transfection reagent. Alternatively, each cell line was incubated in HCVccs (1.4 x 10^6^ infectious units). GFP (Fig 4B) and PTPRE expression (Fig 4C) were monitored 48 hours post transfection or HCVcc incubation using immunoblot and flow cytometry analyses, respectively. HCV gRNA only reduced GFP expression in cells expressing GFP with PTPRE site 2 3’UTR target sequences (Fig 4B). Since site 1 has 38% complementarity and site 2 has 56% complementarity with the HCV E2 vsRNA, these data provide additional support for the hypothesis that the percent complementarity between the vsRNA and PTPRE 3’UTR is critical for gene regulation, and may explain why VAPA and Grb2 were not regulated by HCV RNA. As expected, HCV gRNA and HCVccs reduced PTPRE expression in all three cell lines compared to control cells (Fig 4C). HCVcc’s did not reduce GFP in any of these cell lines, presumably due to the lower concentration of HCV RNA present in this preparation and the high levels of GFP expression.

**Fig 5:**
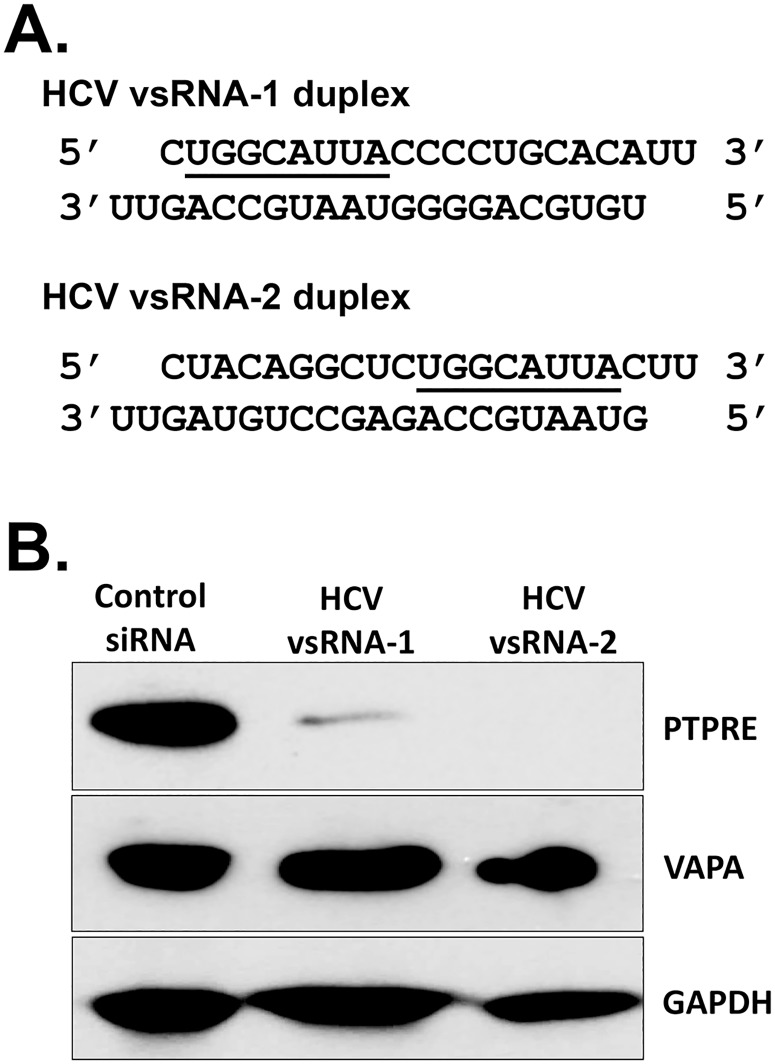
HCV synthetic E2 vsRNAs specifically inhibited PTPRE expression. Huh 7.5 cells were transfected with control siRNA, HCV vsRNA-1 or vsRNA-2 duplexes (putative seed sequence underlined; A). PTPRE, VAPA and GAPDH expression was measured 96 hours post transfection by immunoblot analysis (B). Experiments were performed three times with consistent results.

To further examine RNA-mediated regulation of PTPRE, two RNA duplexes containing the conserved HCV vsRNA targeting sequence were synthesized. The 8 nt HCV sequence was placed at either the 5’ end (vsRNA-1) or the 3’ end (vsRNA-2) of the HCV sequence (Fig 5A). These vsRNAs were transfected into Huh7.5 cells, and both reduced PTPRE but not VAPA levels compared to control cells transfected with non-specific siRNA (Fig 5B), suggesting that the location of the 8 nt seed sequence within the vsRNA may not be critical for PTPRE inhibition.

### PTPRE expression in liver tissue is regulated during human HCV infection

**Fig 6:**
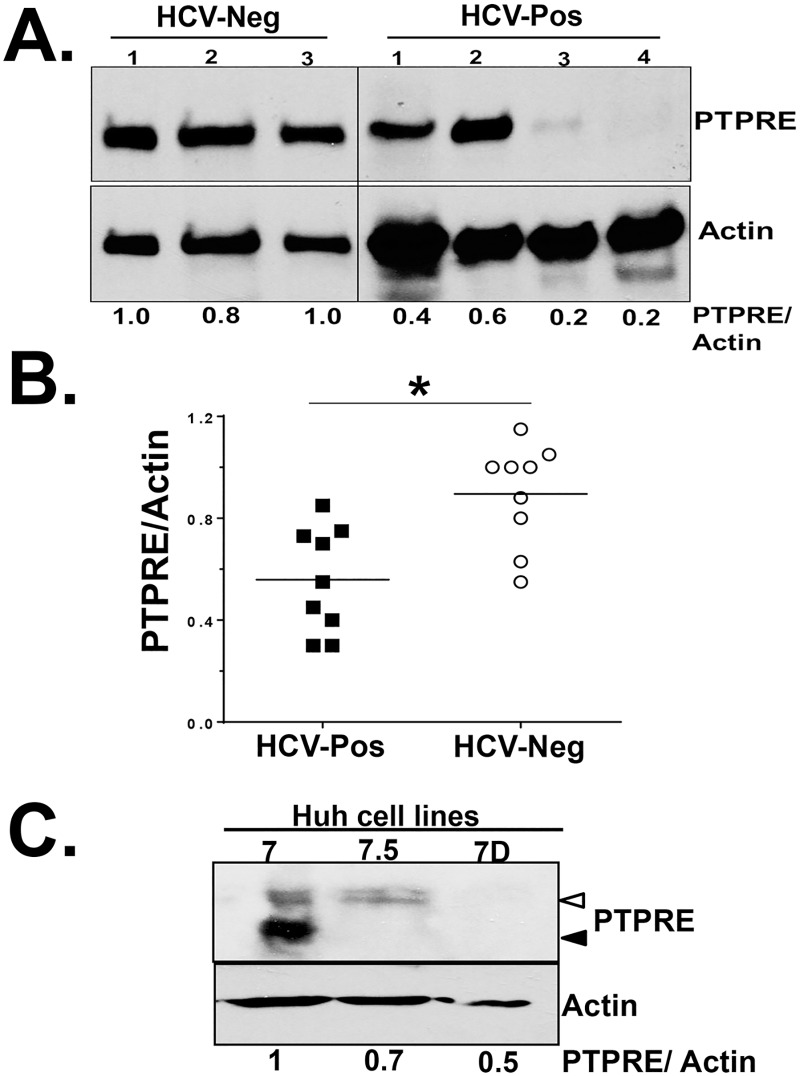
PTPRE expression is reduced in the liver of HCV-infected humans. PTPRE expression in liver explant tissues obtained from HCV-infected and uninfected humans with liver disease were determined by immunoblot analysis (selected examples in panel A). PTPRE levels relative to the actin loading control for all liver tissue samples studied (B). PTPRE levels in the Huh7 hepatoma cell line and in two cell lines clonally selected from Huh7 cells (Huh7.5 and Huh7D). HCV replication is significantly greater in Huh7.5 and Huh7D compared to Huh7 (C). Open arrow = PTPRE isoform 1 (transmembrane), Closed arrow = PTPRE isoform 2 (cytosolic). *P< 0.01.

PTPRE is expressed by hepatocytes in liver tissue and in lymphocytes (web-based protein atlas)(http://www.proteinatlas.org/ENSG00000132334-PTPRE/tissue). HCVgRNA and synthetic HCV vsRNA duplexes are sufficient to reduce PTPRE levels in cells of hepatocyte origin (Figs 2, 3 and 4). Furthermore, serum-derived HCV RNA present in virions or serum extracellular vesicles are transferred into hepatocytes and lymphocytes resulting in reduced PTPRE protein levels and productive infection *in vitro* [11, 44–46]. Since HCV replicates primarily in hepatocytes during human infection [47], we examined PTPRE levels in liver explant tissues obtained from HCV-infected and HCV-uninfected individuals. All liver tissue was evaluated by a pathologist with extensive experience in hepatic pathology, and fibrosis and inflammation scores used the metavir system. Inflammation was graded A0 = no activity, A1 = mild activity, A2 = moderate activity, and A3 = severe activity. Fibrosis was scored as F0 = no fibrosis, F1 = portal fibrosis without septa, F = portal fibrosis with few septa, F3 = numerous septa without cirrhosis, and F4 = cirrhosis. S1 Table summarizes the age, gender, diagnosis, fibrosis and inflammation scores for the subjects. The number of subjects with grade 3 or 4 fibrosis were equal in the HCV and the non-HCV liver tissues (n = 3). PTPRE levels were significantly lower in tissue obtained from HCV-infected humans compared to liver tissues from people with liver disease other than HCV infection when normalized to GAPDH (Fig 6A and 6B), and PTPRE levels did not correlate with inflammation or fibrosis score (Fibrosis data shown in S1 Fig).

Since PTPRE activates Src-kinases, and previous studies found an inverse relationship between HCV replication and Src-kinase signaling [24, 25], PTPRE may be a previously unrecognized viral restriction factor in HCV infection. Interestingly, PTPRE levels were lower in Huh7.5 and Huh7D human hepatoma cell lines compared to the Huh7 cell line that they were clonally derived from (Fig 4C)[48], and HCV replicates significantly higher in Huh7.5 and Huh7D cells compared to the parental Huh7 cell line [48]. Early studies suggested that a mutation in RIG-I in Huh7.5 cells may contribute to enhanced HCV replication; however, subsequent studies found that Huh7D cells do not have the RIG-I mutation, yet support HCV replication as well as Huh7.5 cells [48]. PTPRE variant 1 is a transmembrane protein while PTPRE variant 2 lacks the transmembrane sequence, and is strictly cytoplasmic [49]. Both Huh7.5 and Huh7D cell lines had lower levels of both PTPRE variant-1 (transmembrane, open arrow) and variant-2 (cytosolic, closed arrow) compared to Huh7 cells (Fig 4C). Thus, there is an association between reduced PTPRE levels and HCV replication in hepatoma cell lines *in vitro* and PTPRE levels are reduced in liver tissue from HCV infected people compared to HCV uninfected, suggesting that PTPRE may interfere with HCV replication by promoting Src-kinase signaling.

### PBMC PTPRE and TCR stimulated Lck activation are reduced by HCV infection

HCV RNA is present in, or bound to PBMCs and platelets [50–52], and HCV infection is associated with impaired IL-2 and IFN-γ responses following stimulation [53]. Incubation of healthy donor PBMCs in HCV RNA-containing particles leads to reduced IL-2 release and surface expression of T cell activation markers [11]. Thus, we examined PTPRE expression in lymphocytes obtained from HCV-infected individuals before and following curative HCV therapy, and compared the results with PTPRE levels in HCV uninfected subjects. S2 Table summarizes the age, gender, diagnosis, fibrosis, and inflammation scores for the subjects.

**Fig 7:**
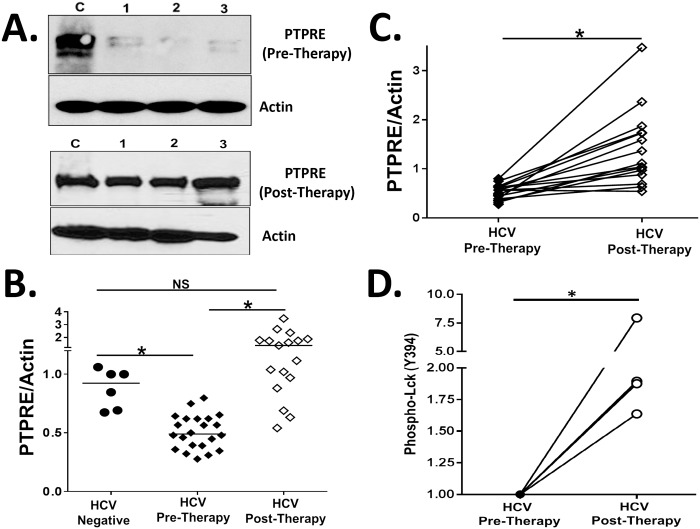
PTPRE expression is reduced in Peripheral Blood Mononuclear Cells (PBMCs) obtained from HCV-infected humans and restored following curative HCV therapy. PTPRE levels in PBMCs obtained from three HCV-infected individuals (1, 2, 3) and uninfected control (C) subjects before and following direct acting antiviral HCV therapy (A) as determined by immunoblot analysis. PTPRE expression relative to actin in the PBMCs obtained from healthy blood donors, and HCV-infected subjects before or following curative HCV therapy (B). PTPRE levels relative to actin in PBMCs in individual HCV-infected subjects before and following HCV therapy (C). PBMCs from four HCV-infected subjects prior to and following HCV therapy were stimulated with anti-CD3. Lck activation (Y394 phosphorylation) was measured and normalized to GAPDH (D). *P< 0.01.

PTPRE expression was significantly lower in HCV-infected PBMCs compared to controls, and rose to levels comparable or higher than HCV uninfected controls following curative HCV therapy (Fig 7A–7C).

Lck activation (phosphorylation of Y394) following TCR stimulation is required for TCR-mediated activation and proliferation [54]. We examined the ability of anti-CD3 stimulation to phosphorylate Lck in PBMCs from subjects before and after HCV therapy. Lck phosphorylation following 5 minutes TCR stimulation was significantly higher in subjects cured of HCV by therapy compared to pre-treatment levels in the same subjects (Fig 7D). These findings are consistent with recent studies demonstrating that curative anti-HCV therapy restores immune cell function in HCV-infected humans by other measures [55, 56].

**Fig 8:**
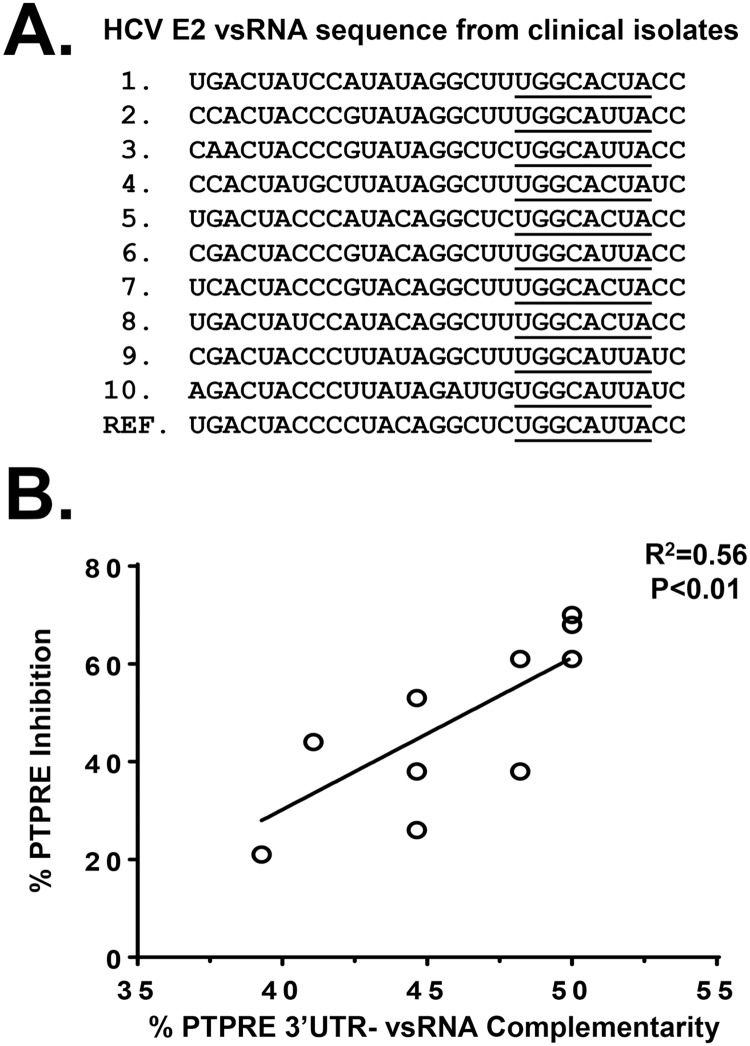
HCV vsRNA sequence polymorphisms correlated with PTPRE inhibition *in vivo*. HCV E2 RNA was amplified from plasma obtained from ten HCV-infected subjects and the vsRNA region was sequenced (A). The putative “seed” sequence for PTPRE is underlined. PTPRE expression levels correlated with the percent complementarity between the HCV vsRNA and the two PTPRE 3’UTR target site sequences (Spearman correlation used to calculate R and P values, and results of best-fit linear regression analysis are shown). Ref = reference sequence from infectious HCV clone.

Andrew Fire’s group identified HCV vsRNAs in HCV-infected Huh7.5 cells [31], one of which was the vsRNA we identified that reduces PTPRE protein expression *in vitro* (vsRNA sequences kindly provided by Drs. Fire and Parameswaran)[11]. Since PBMC PTPRE levels varied somewhat among HCV-infected individuals (as in Fig 7B), we sequenced the HCV E2 RNA present in serum obtained from ten subjects to determine if there is a relationship between PTPRE expression and HCV sequence diversity (Fig 8A, underlined) [31]. Examining the patient’s 29 base E2 sequence that is detected as a vsRNA in HCV-infected cells [11], the 8 nt region complementary to PTPRE 3’UTR was highly conserved. However, there were numerous sequence polymorphisms in the flanking sequences (Fig 8A). To quantify this, the percent of bases in the HCV vsRNA sequences from each of the ten subjects complementary to the two PTPRE 3’UTR target sequences were correlated with the expression of PTPRE in their PBMCs (Fig 8B). The greater the percent complementarity between each subjects’ E2 RNA sequence with the PTPRE 3’ UTR, the lower the level of PTPRE expression detected (R^2^ 0.56, p<0.01 Spearman Correlation).

## Discussion

Virus derived small RNAs (vsRNAs) encoded by DNA viruses and retroviruses play an important role in viral replication and may contribute to immune evasion [26, 27]. Although vsRNAs are found in cells infected with cytoplasmic RNA viruses *in vitro* [11, 31–35, 57, 58], their role in human infection is not characterized, and their significance is debated [28, 30]. HCV is unusual among cytoplasmic RNA viruses in that it establishes persistent infection in the majority of infected people [2, 59]. Previous studies found that HCV infection impairs T cell function, and presumably this contributes to viral persistence [7–11, 59].

Although several mechanisms may contribute to HCV immune evasion, we recently found that expression of full-length HCV E2 coding RNA with a frame-shift to abolish translation in Jurkat cells reduced TCR signaling and Lck activation following TCR stimulation with anti-CD3/CD28 [11]. Placing the PTPRE 3’UTR after GFP, the expression of HCV E2 RNA regulated GFP in transient transfection experiments, and expression of the RNA with 4 bases substituted restored TCR activity and PTPRE levels. Finally, when the sequence that is complementary to the PTPRE 3’UTR was replaced with a sequence targeting CXCR4, the RNA reduced CXCR4 and not PTPRE [11]. We also demonstrated that HCV RNA-containing plasma, HCVccs, and plasma-derived HCV RNA-containing micro-vesicles impaired IL-2 release by Jurkat cells and primary human PBMCs and purified T cells following stimulation with anti-CD3 antibody [11].

Here, we expand the earlier findings to show that HCV containing serum inhibits both antigen-specific (CEF-mediated) TCR signaling, and PTPRE expression, and that T cell function and PTPRE levels are restored following curative HCV therapy, providing novel insights into antigen-specific and non-specific modulation of T cells by HCV RNA. We also demonstrated that *in vitro* transcribed full-length HCV genome and short synthetic HCV E2 vsRNAs were sufficient to regulate PTPRE expression following transfection into Huh7.5 cells, and that one of the two complementary sequences in the PTPRE 3’UTR is sufficient to regulate upstream protein expression. We also observed that PTPRE levels were reduced in liver biopsy tissues and cell lines of hepatocyte origin that support HCV replication, and that HCV infection was associated with both reduced PTPRE levels in PBMCs, and with reduced Lck activation following TCR stimulation. PTPRE levels and Lck phosphorylation were restored by curative HCV therapy. Further supporting a functional role for the HCV vsRNA, the extent of sequence complementarity between the HCV E2 RNA sequences correlated directly with the level of reduction of PTPRE expression in lymphocytes. Although complete blockade of TCR signaling would render an infected person severely immune compromised, HCV effects on TCR signaling are incomplete. This regulator of TCR signaling likely contributes to both establishment of infection and persistent viremia.

Although there are many vsRNAs detected in HCV infected cells for which no function has been determined [31], these data suggest that vsRNAs may serve as an unexplored mechanism of HCV regulation of host gene expression. Nevertheless, several important questions remain. For example, how does HCV RNA interact with and regulate cellular gene targets? We speculate that the HCV RNA-containing virions, lipo-particles, and exosomes released from the liver during viral replication deliver viral RNA to uninfected hepatocytes and lymphocytes. Early studies showed that hepatic lymphocytes are more impaired in proliferative activity than circulating lymphocytes [8], and we propose that this may be explained by the greater exposure of hepatic lymphocytes to HCV RNA. Given that plasma HCV RNA concentrations are typically > 1 million copies/ml, circulating lymphocytes are also exposed to HCV RNA-containing particles [11]. Another question relates to the concentration of viral RNA and whether it is sufficient to regulate cellular genes [28]. It is important to note that standard methods to detect HCV RNA will not detect vsRNAs, and thus are likely to underestimate the concentration of small RNA present in plasma and lymphocytes. The findings reported here clearly demonstrate that HCV RNA regulates PTPRE and Lck activation *in vitro*, and that HCV infection regulates TCR activation and PTPRE expression *in vivo*.

Among potential targets identified by bioinformatics, VAPA, a proviral factor required for HCV replication [39–42], and Grb 2 were not reduced by HCV vsRNA despite having putative binding sites in their 3’UTRs. The extent of complementarity between the HCV vsRNA and the VAPA and Grb2 3’ UTRs was the same as the PTPRE site 1 (38%). Neither VAPA nor Grb2 were regulated by HCV RNA, and placing PTPRE site 1 downstream of GFP did not lead to downregulation of GFP, suggesting that sequence diversity outside the conserved seed sequence that reduced the amount of complementarity with the potential target sequences is critical for the specificity of target gene regulation. Further supporting this hypothesis, HCV RNA-containing sera reduced GFP expression when PTPRE site 2 was placed downstream of the GFP coding region, and the extent of PTPRE reduction correlated with the complementarity between the HCV E2 RNA sequence detected in clinical isolates and the PTPRE 3’UTR (Fig 8B).

Our data also identified PTPRE as a novel factor regulated by HCV vsRNA in hepatocytes. This phosphatase activates Src-kinase signaling, and previous studies demonstrate an inhibitory role of Src-kinases in HCV replication [24, 25]. Thus, vsRNA mediated inhibition of PTPRE expression in hepatocytes may promote viral replication in addition to contributing to T cell dysfunction. In summary, these data indicate that PTPRE plays an important role in T cell function and potentially HCV replication, and may serve as an attractive target for anti-HCV or immunomodulatory therapeutics.

## Methods

### Study subjects

HCV-infected subjects recruited from the University of Iowa Hepatology Clinic or healthy blood donors were invited to participate in this study. Characteristics of subjects are described in S2 Table. Liver biopsy protein was extracted by sonication in protein extraction buffer (Tris-HCl, NP-40, NaCl, EDTA, protease inhibitors, pH 7.5), and cellular lysate protein concentrations were determined by Pierce BCA Protein Assay Kit.

### Cells and viruses

Human cell lines Huh7 cells (obtained from the American Type Culture Collection) Huh-7.5 (kindly provided by Dr. Charles Rice), and Huh7D (kindly provided by Dr. Dino Feigelstock), were cultured in Dulbecco's modified Eagle's medium containing 10% fetal bovine serum, 1% penicillin-streptomycin and 1% L-glutamine at 37°C in a 5% CO_2_. HCV genomic RNA (gRNA) was transcribed from J6/JFH infectious clone as described [43]. PBMC isolation was performed as previously described [11, 60].

### GFP expression regulation by PTPRE 3’UTR sequences

Coding sequence for eGFP were ligated into a modified pTRE2-HGY plasmid (Clontech, Inc.) expressing GFP with an EMC IRES element directing translation as previously described [61]. The two putative target sites within the PTPRE 3’UTR were inserted after the GFP open reading frame (Site 1 = TGCAGTTGGGTTCAAATGCCAAATAGTGATTAGAAGACGA (38% complementary to HCV vsRNA); Site 2 = ATAGTGTTCGACTTCAAATGCCACGACGCGGCCG (56% complementary to HCV vsRNA) and PTPRE sequences were confirmed by sequencing plasmid DNA (University of Iowa DNA Core Facility). Jurkat (tet-off) cell lines (Clontech, Inc) were transfected (Nucleofector II, Lonza Inc.) and cell lines were selected for hygromycin and G418 resistance. GFP positive cells were bulk sorted (BD FACS Aria, (University of Iowa Flow Cytometry Facility) and GFP expression was assessed by flow cytometry (BD LSR II). All cell lines were maintained in RPMI 1640 supplemented with 10% heat-inactivated fetal calf serum, 2mM L-glutamine, 100 IU/ml penicillin, and 100 μg/ml streptomycin with hygromycin and G418 (200 μg/ml).

### T cell receptor-mediated activation

PBMCs (2×10^6^ cells/ml) obtained from HCV-negative donors were resuspended in 200 μl serum obtained from HCV-infected donors before or after curative HCV therapy and incubated for 24 hrs prior to stimulation with plate-bound anti-CD3 (100 ng/ml, OKT3 clone, eBioscience). Alternatively, antigen-specific TCR-mediated activation was stimulated using pooled synthetic peptides (20 μg/ml) with sequences derived from human cytomegalovirus, Epstein-Barr virus, and influenza viruses (CEF control peptides, AnaSpec, EGT Group)[62, 63]. TCR-mediated signaling was determined 16 hours post-TCR stimulation by measuring IL-2 using ELISA as described [64, 65], or by measuring activated Lck protein as described below. Each experiment was performed in three replicate cultures, and in a minimum of three healthy donor PBMCs with consistent results.

### HCV E2 sequencing

Plasma RNA was isolated from HCV-infected humans (QIAmp Viral RNA Kit, Qiagen) and cDNA generated using random hexamer primers as described [66]. HCV E2 was amplified using either genotype specific or degenerate primers: Sense 5’-WCDGGHCAYCGMATGGCD TGGGA and antisense 5’-GCAGAAGAACACGAGGAAGGASA. PCR products were cloned into the TA cloning vector pCR2.1 (Invitrogen) and automated DNA sequences obtained by the University of Iowa DNA Core Facility [67].

### HCV RNA transfection

Huh7.5 cells were transfected by electroporation (Bio-Rad Gene Pulser Xcell) using 10μg HCV genomic RNA (gRNA). HCV and control vsRNA duplexes were purchased from Integrated DNA Technologies and used at 1μM concentrations. Cells and HCV RNA were mixed in cold PBS (500μL) transferred to a 4 mm gap-width electroporation cuvette and pulsed once at 270V and 950 μF capacitance. Transfected cells were maintained in complete medium for 96 hours at 37°C.

### Immune blot analyses

Cell lysates were separated by SDS-PAGE gel electrophoresis, transferred to nitrocellulose membranes, and proteins detected by chemiluminescence as described [65, 68]. Primary antibodies included phospho-Lck Y394 (R&D Systems), PTPRE (4B2) and GAPDH (Origene), PTPRE (Rabbit; Abcam), or Actin (Sigma). Immunoblots were quantified using ImageJ.

### Statistics

Statistics were performed using GraphPad software V4.0 (GraphPad Software Inc.). Student’s t test was used to compare results between groups. *P* values less than 0.05 were considered statistically significant.

### Ethics statement

This study was approved by the University of Iowa Institutional Review Board (IRB-01) and all subjects provided written informed consent.

## Supporting information

S1 Fig PTPRE expression did not correlate with fibrosis score. Although the HCV infected subjects were shown to have lower PTPRE expression relative to actin, there was no correlation observed between PTPRE expression levels and fibrosis score in either HCV infected or HCV uninfected subjects. ND = no data as there were no subjects with HCV with no fibrosis detected on biopsy.
(TIF)

S1 Table Characteristics of subjects with liver biopsy tissues. ALD = alcoholic liver disease; HH = hereditary hemochromatosis; LAE = liver enzyme elevation; NASH = non-alcoholic steato-hepatitis; PSC = primary sclerosing cholangitis; RCC = renal cell carcinoma with elevated liver enzymes; Tx = liver transplant organ biopsy. *PTPRE relative to Actin by immune blot.
(DOCX)

S2 Table Characteristics of HCV-infected subjects. L = Ledipasvir; S = Sofosbuvir; R = Ribavirin; V = Ombitasvir+ Paritaprevir+ Ritonavir+ Dasabuvir.
(DOCX)
