# Supplementary figures and images for: Hepatitis C virus infection inhibits a Src-kinase regulatory phosphatase and reduces T cell activation in vivo
Source: PLoS Pathog. 2017 Feb 24;13(2):e1006232. doi: 10.1371/journal.ppat.1006232 (PMC5342304; doi:10.1371/journal.ppat.1006232)

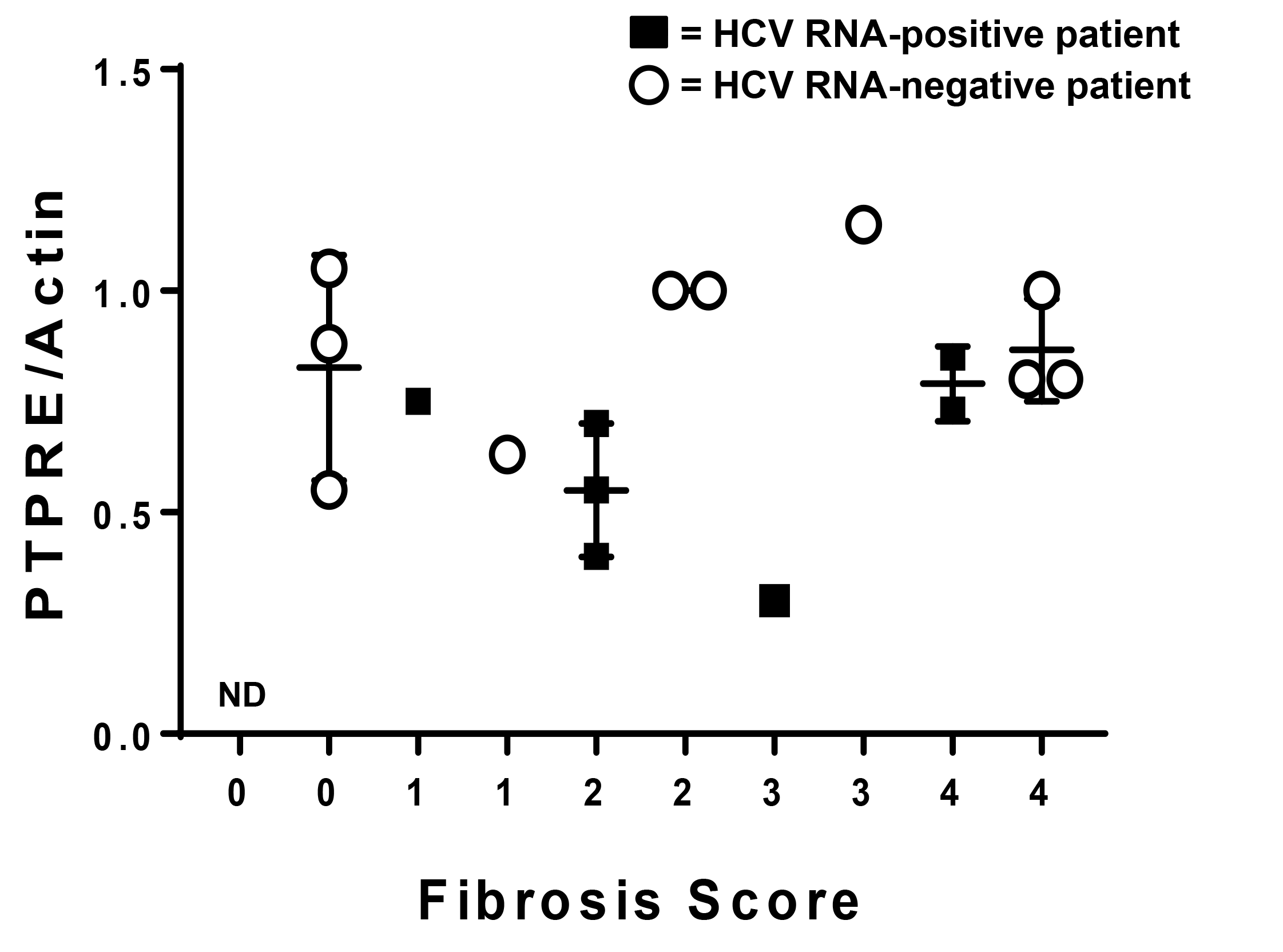

Supplement: S1 Fig — Although the HCV infected subjects were shown to have lower PTPRE expression relative to actin, there was no correlation observed between PTPRE expression levels and fibrosis score in either HCV infected or HCV uninfected subjects. ND = no data as there were no subjects with HCV with no fibrosis detected on biopsy. (TIF) [file ppat.1006232.s001.tif]
