# Supplementary material for: Hepatitis C virus infection inhibits a Src-kinase regulatory phosphatase and reduces T cell activation in vivo
Source: PLoS Pathog. 2017 Feb 24;13(2):e1006232. doi: 10.1371/journal.ppat.1006232 (PMC5342304; doi:10.1371/journal.ppat.1006232)
Supplement: S1 Table — ALD = alcoholic liver disease; HH = hereditary hemochromatosis; LAE = liver enzyme elevation; NASH = non-alcoholic steato-hepatitis; PSC = primary sclerosing cholangitis; RCC = renal cell carcinoma with elevated liver enzymes; Tx = liver transplant organ biopsy. *PTPRE relative to Actin by immune blot. (DOCX) [file ppat.1006232.s002.docx]

**S1 Table. Characteristics of subjects with liver biopsy tissues**

| Subject | Age | Gender | Diagnosis | Fibrosis | Inflammation | PTPRE expression* |
| --- | --- | --- | --- | --- | --- | --- |
| 1 | 67 | M | HCV | 4 | 2 | 0.73 |
| 2 | 46 | M | HCV | 2 | 3 | 0.4 |
| 3 | 50 | M | HCV | 3 | 3 | 0.3 |
| 4 | 49 | F | HCV | 1 | 1 | 0.75 |
| 5 | 53 | M | HCV | 4 | 2 | 0.85 |
| 6 | 58 | M | HCV | 0 | 1 | 0.45 |
| 7 | 57 | F | HCV | 2 | 1 | 0.7 |
| 8 | 48 | M | HCV | 2 | 3 | 0.55 |
| 9 | 41 | N | HCV | 1 | 0 | 0.3 |
| 10 | 63 | F | PSC | 2 | 1 | 1.0 |
| 11 | 45 | M | ALD | 4 | 3 | 0.8 |
| 12 | 55 | M | ALD | 3 | 1 | 1.15 |
| 13 | 43 | F | PSC | 2 | 0 | 1.0 |
| 14 | 56 | M | ALD/Tx | 1 | 2 | 0.63 |
| 15 | 40 | M | RCC | 0 | 3 | 1.05 |
| 16 | 52 | M | HH/Tx | 0 | 0 | 0.55 |
| 17 | 46 | M | LAE | 0 | 2 | 0.88 |
| 18 | 41 | F | ALD | 4 | 3 | 1.0 |

ALD = alcholic liver disease; HH = hereditary hemochromatosis; LAE = liver enzyme elevation; NASH = non-alcoholic steato-hepatitis; PSC = primary sclerosing cholangitis; RCC = renal cell carcinoma with elevated liver enzymes; Tx = liver transplant organ biopsy. *PTPRE relative to Actin by immune blot
