# Supplementary material for: Hepatitis C virus infection inhibits a Src-kinase regulatory phosphatase and reduces T cell activation in vivo
Source: PLoS Pathog. 2017 Feb 24;13(2):e1006232. doi: 10.1371/journal.ppat.1006232 (PMC5342304; doi:10.1371/journal.ppat.1006232)
Supplement: S2 Table — L = Ledipasvir; S = Sofosbuvir; R = Ribavirin; V = Ombitasvir+ Paritaprevir+ Ritonavir+ Dasabuvir. (DOCX) [file ppat.1006232.s003.docx]

**S2 Table: Characteristics of HCV-infected subjects**

| **I.D.** | **Age (years)** | **Gender** | **HCV VL** | **Genotype** | **Prior IFN** | **Med** | **Duration (weeks)** |
| --- | --- | --- | --- | --- | --- | --- | --- |
| 1 | 57 | M | 3.89E+06 | 1a | No | L+S | 12 |
| 2 | 59 | M | 1.31E+06 | 1b | Yes | L+S | 12 |
| 3 | 54 | M | 2.45E+05 | 1b | No | V+R | 12 |
| 4 | 66 | M | 1.95E+06 | 1b | No | L+S | 12 |
| 5 | 61 | M | 5.50E+06 | 1a | No | L+S | 12 |
| 6 | 57 | M | 6.76E+06 | 1 | Yes | L+S | 24 |
| 7 | 61 | M | 5.01E+06 | 1b | No | L+S | 12 |
| 8 | 60 | F | 3.72E+06 | 1a | No | L+S | 12 |
| 9 | 60 | F | 4.17E+05 | 1a | No | L+S | 12 |
| 10 | 59 | M | 6.03E+06 | 2 | No | S+R | 16 |
| 11 | 68 | M | 1.95E+06 | 3 | Yes | S+R | 24 |
| 12 | 39 | M | 7.76E+05 | 1a | No | L+S | 12 |
| 13 | 64 | F | 1.02E+06 | 1 | Yes | L+S | 12 |
| 14 | 61 | M | 8.71E+05 | 1 | No | L+S | 24 |
| 15 | 58 | F | 3.63E+05 | 1 | No | L+S+R | 24 |
| 16 | 56 | M | 1.78E+06 | 1 | No | L+S | 24 |
| 17 | 54 | M | 1.98E+06 | 1a | Yes | L+S | 12 |
| 18 | 52 | M | 7.76E+04 | 1a | No | L+S | 12 |
| 19 | 52 | M | 1.62E+05 | 1 | Yes | L+S | 24 |
| 20 | 58 | F | 3.63E+06 | 1a | No | L+S+R | 12 |
| 21 | 65 | F | 1.70E+06 | 1a | YES | L+S | 12 |
| 22 | 51 | F | 1.49E+06 | 1 | No | L+S | 24 |

L= Ledipasvir; S=Sofosbuvir; R= Ribavirin; V= Ombitasvir+ Paritaprevir+ Ritonavir+ Dasabuvir
